# ResearchGate as a strategic tool to enhance the scientific visibility in Speech-Language Pathology and Audiology

**DOI:** 10.1590/2317-1782/e20250158en

**Published:** 2026-01-26

**Authors:** Vanessa Luisa Destro Fidêncio

**Affiliations:** 1 Programa de Pós-Graduação em Saúde da Comunicação Humana – PPGSCH, Universidade Tuiuti do Paraná – UTP - Curitiba (PR), Brasil.

## Dear editors of the CoDAS Journal,

This letter aims to propose a reflection on the strengthening of the presence of Speech-Language Pathology (SLP) and Audiology researchers in the digital environment, especially in the ResearchGate academic social network, in order to broaden the scope and impact of research developed in the area.

In an editorial published in 2020, Navas et al.^(1)^ discussed the democratization of the technical-scientific knowledge in the digital environment, by means of social networks as a tool to reduce the gaps between research and clinical practice, as well as the fast, accessible spread of the scientific content. Additionally, in a second editorial, published in 2024^(2)^, the authors discussed the impact of COVID-19 pandemic on scientific dissemination by collecting data of CoDAS Journal on Instagram, Twitter, Facebook and LinkedIn networks. Other confirmations^(3,4)^ have placed Brazilian researchers among those who believe the most in the ResearchGate potential for academic visibility, and those who have the greatest intention to increase their activity in the network. However, due to the scarcity of publications about it, that network has still been little explored by SLP and Audiology researchers in Brazil.

ResearchGate^(5)^, founded in 2008, is a social network exclusively dedicated to the professional use by the scientific community^(6)^. Its members may add data such as academic degree, institutional affiliations, performance areas, educational background, skills, awards, scholarships, funding, partnerships with scientific societies, ORCID number, and roles performed in scientific periodicals. In addition, they share their academic productions, such as articles published in periodicals. Their profile can be viewed by members of the platform and, configured as a public one, it can also be viewed by non-members and indexed by search engines^(7)^.

ResearchGate matches traditional elements and new approaches regarding building and monitoring of academic reputation, entailing a broad variety of metrics. Certain metrics, such as the number of reads – internally calculated by the system – are updated almost in real time. Additionally, notifications are e-mailed whenever its members have their works read, followed or cited, enabling ongoing follow-up of their visibility and academic impact^(4)^. Questions and answers (Q&A) intend to enhance network engagement, promoting spaces where its members can ask questions and obtain answers^(4,8)^. Finally, one of the metrics used in ResearchGate is the ResearchGate score (RG *score*). Despite its real composition is unknown, this metrics is assumed to entail the following dimensions: publications (50%), Q&A (comprising 25% for answers and 24% for questions), and followers (1%)^(8)^.

The first study, which aimed to analyze impact metrics of SLP and Audiology researchers in ResearchGate, dates from 2017^(9)^. Over 2,000 faculty members from 257 accredited programs in the SLP and Audiology area were assessed in the USA and Canada. The faculty members entailed the Audiology area (24.4%; n=490) and the Speech Therapy area (75.6%; n=1.520). Females accounted for 68.1% of the Faculty, while males accounted for 31.9%. Faculty ratio with profile in the ResearchGate comprised 44% (n=885). Three network metrics were assessed: number of publications, number of citations and RG *score*. Faculty members in the Audiology area showed significantly higher median values in all metrics. Such differences were also observed between males and females, with higher medians verified among males. In addition, indexes increased progressively along the academic career.

Social networks have become powerful, essential tools for the scientific community^(10)^, and ResearchGate has potential to boost scientific collaboration and promote knowledge advancement^(11)^. However, some metrics have still been controversial, such as the RG score^(6)^. The RG *score* considers the Q&A tool, and evidence^(8,12)^ uncovers little interest in its use by the network members. Moreover, gender issues may influence in the reputation metrics of its signed-up members^(9,10)^. Therefore, experts point out the following metrics as the most valuable to assess the reputation of Speech-Language Therapy and Audiology researchers: number of published scientific productions (total number and from the last five years), and number of citations, excluding self-citations (total number and from the last five years)^(9)^.

Although the huge benefits of social networks like ResearchGate should be acknowledged, it is also essential to recognize the implied challenges^(13)^. It should be highlighted here the related risk of copyright violation. On ResearchGate, researchers may share their scientific productions in different formats such as, full text, abstracts and PDF files. However, when an article is published in a scientific peer-reviewed periodical, it is common for the authors to sign a contract with the publishing company, scientific society or the periodical itself, granting exclusivity for publication. Thus, this kind of contract prevents sharing of the full text by other platforms or networks. In such cases, the authors can only include the official link of the article on the site of the periodical, which may be or not freely accessed, depending on the access policies of the publication. Even in open-access periodicals, the recommendation is that the researcher only shares the official link on the ResearchGate, preventing the direct sending of the full PDF^(14)^.

ResearchGate is an emerging network, still in progress, whose metrics can still be modified^(4)^. As profile registration and keeping on this network are voluntary, researchers with greater scientific production are likely to be more motivated to build and make their profiles public for visibility and recognition. On the other hand, those with lower bulk of publications may opt for not joining that network, which may lead to underrepresentation of that group. As a result, author indicators may show inflated figures, reflecting the engaged members’ profile rather than the actual, complete picture of the academic productivity in the area^(9)^.

It is understood that the analysis of quantitative measurements for the scientific impact, at the author’s level, is justified in the SLP and Audiology area. Motivation may vary according to the context: whether it is a faculty member reviewing his/her own or other’s production, a manager making decisions on hiring, tenure or promotion; or even a development agency evaluating funding requests^(9)^. ResearchGate transcends geographic borders, enabling researchers to connect and collaborate with their peers around the world. It promotes worldwide research efforts, interdisciplinary collaboration and exchange of good practices and innovative ideas^(11)^. After all, it will be utterly necessary to promote actions of awareness and qualification so that researchers realize the challenges related to the use of social networks and the reasons why they should be accessed^(15)^. In this scenario, the strategic use of the ResearchGate is highlighted, whose interface, targeting the scientific community, not only enhances the visibility of Speech-Language Therapy and Audiology productions, but it also strengthens collaborations, real-time spread of knowledge, and the construction of a more open, connected scientific culture, aligned with the contemporary demands of Science promotion and accessibility.
