# Supplementary material for: ResearchGate as a strategic tool to enhance the scientific visibility in Speech-Language Pathology and Audiology
Source: Codas. 2026 Jan 26;38(1):e20250158. doi: 10.1590/2317-1782/e20250158en (PMC12978328; doi:10.1590/2317-1782/e20250158en)
Supplement: Translation

# Translation: A ResearchGate como ferramenta estratégica para a visibilidade científica na Fonoaudiologia

**DOI:** 10.1590/2317-1782/e20250158pt

**Authors:** Vanessa Luisa Destro Fidêncio

**Affiliations:** 1 Programa de Pós-Graduação em Saúde da Comunicação Humana – PPGSCH, Universidade Tuiuti do Paraná – UTP - Curitiba (PR), Brasil.

## Prezadas editoras da Revista CoDAS,

A proposta desta carta é propor uma reflexão sobre o fortalecimento da presença de pesquisadores(as) fonoaudiólogos(as) no ambiente digital, com ênfase na rede social acadêmica *ResearchGate*, a fim de ampliar o alcance e o impacto das pesquisas desenvolvidas na área.

Em um editorial publicado em 2020, Navas et al.^(1)^ discutiram a democratização do conhecimento técnico-científico no ambiente digital, com uso das redes sociais como ferramenta para diminuir as distâncias entre a pesquisa e a prática clínica e disseminação rápida e acessível do conteúdo científico. Já em um segundo editorial, publicado em 2024^(2)^, os autores discutiram a respeito dos impactos da pandemia de COVID-19 para a divulgação científica, levantando dados do perfil da revista CoDAS nas redes sociais *Instagram*, *Twitter*, *Facebook* e *LinkedIn*. Outras evidências^(3,4)^ colocaram os pesquisadores brasileiros entre os que mais acreditam no potencial da *ResearchGate* para a visibilidade acadêmica e com maior intenção de aumentar a atividade na rede. No entanto, pela falta de publicações a respeito, acredita-se que essa seja ainda uma rede pouco explorada por pesquisadores da área da Fonoaudiologia no Brasil.

A *ResearchGate* ^(5)^, fundada em 2008, trata-se de uma rede social dedicada exclusivamente ao uso profissional da comunidade científica^(6)^. Os membros podem adicionar dados como grau acadêmico, afiliações institucionais, áreas de atuação, histórico educacional, habilidades, prêmios, bolsas, financiamentos, vínculos com sociedades científicas, número ORCID e papéis desempenhados em periódicos científicos. Além disso, compartilham suas produções acadêmicas, como artigos publicados em periódicos. O perfil é pesquisável por membros da plataforma e, se configurado como público, também pode ser encontrado por não-membros e indexado por mecanismos de busca^(7)^.

A *ResearchGate* combina elementos tradicionais com novas abordagens no que se refere à construção e ao monitoramento da reputação acadêmica, com ampla variedade de métricas. Determinadas métricas, como o número de leituras (*reads*) — calculado internamente pelo sistema — são atualizadas em tempo quase real. Além disso, são enviadas notificações por e-mail sempre que um membro tem seus trabalhos lidos, seguidos ou citados, permitindo o acompanhamento contínuo de sua visibilidade e impacto acadêmico^(4)^. As perguntas e respostas (Q&A) têm a intenção de aumentar o engajamento na rede, promovendo espaços onde os membros podem fazer suas perguntas e obter respostas^(4,8)^. Por fim, uma das métricas utilizadas na *ResearchGate* é o *ResearchGate score* (RG *score*). Apesar de sua composição real ser desconhecida, hipotetiza-se que essa métrica seja composta pelas seguintes dimensões: publicações (50%), Q&A (sendo 25% para respostas e 24% para perguntas) e seguidores (1%)^(8)^.

O primeiro estudo que se propôs a analisar métricas de impacto de pesquisadores fonoaudiólogos na *ResearchGate* data de 2017^(9)^. Foram avaliados mais de 2000 docentes de 257 programas credenciados na área da Fonoaudiologia nos EUA e Canadá. Os docentes estavam distribuídos entre a área de Audiologia (24,4%; n=490) e a de Terapia da Fala (75,6%; n=1.520). As mulheres representavam 68,1% do corpo docente, enquanto os homens correspondiam a 31,9%. A proporção de docentes com perfil na *ResearchGate* foi de 44% (n=885). Foram analisadas três métricas da rede: número de publicações, número de citações e o RG *score*. Docentes da área de Audiologia apresentaram valores medianos significativamente mais altos em todas as métricas. Essas diferenças também foram observadas entre homens e mulheres, com maiores medianas entre os homens. Além disso, os índices aumentaram progressivamente com o avanço na carreira acadêmica.

As redes sociais tornaram-se ferramentas poderosas e essenciais para a comunidade científica^(10)^ e a *ResearchGate* apresenta potencial para aumentar exponencialmente a colaboração científica e promover o avanço do conhecimento^(11)^. No entanto, algumas métricas ainda apresentam-se controversas, como o RG *score* ^(6)^. O RG *score* considera a ferramenta Q&A e evidências^(8,12)^ apontam que há pouco interesse no uso desta por grande parte dos membros cadastrados na rede. Além disso, questões de gênero podem influenciar nas métricas de reputação dos pesquisadores cadastrados^(9,10)^. Assim, especialistas referem como mais valiosas na análise da reputação de pesquisadores na Fonoaudiologia as seguintes métricas: número de produções científicas publicadas (total e nos últimos cinco anos) e número de citações excluindo autocitações (total e nos últimos cinco anos)^(9)^.

Embora se reconheçam os imensos benefícios de redes sociais como a *ResearchGate*, é crucial reconhecer os desafios associados^(13)^. Destacam-se aqui os riscos relacionados à violação de direitos autorais. Na *ResearchGate*, os pesquisadores podem compartilhar suas produções científicas em diferentes formatos, como texto completo, resumos e arquivos em PDF. No entanto, quando um artigo é publicado em um periódico científico com revisão por pares, é comum que o autor tenha firmado um contrato com a editora, sociedade científica ou com o próprio periódico, concedendo exclusividade para a publicação. Em geral, esse tipo de contrato impede o compartilhamento do texto completo em outras plataformas ou redes. Nesses casos, os autores podem incluir apenas o *link* oficial para o artigo no site do periódico, que pode ou não estar disponível gratuitamente, dependendo das políticas de acesso da publicação. Mesmo nos periódicos de acesso aberto, a recomendação é que o pesquisador compartilhe na *ResearchGate* apenas o *link* oficial, evitando o envio direto do PDF completo^(14)^.

A *ResearchGate* é uma rede emergente, que ainda está em progresso, cujas métricas ainda poderão ser modificadas^(4)^. Como o registro e a manutenção de perfis nessa rede são voluntários, é plausível que pesquisadores com maior produção científica se sintam mais motivados a criar e tornar públicos seus perfis, buscando visibilidade e reconhecimento. Em contrapartida, aqueles com menor volume de publicações podem optar por não aderir a essa rede, o que gera uma sub-representação desse grupo. Como resultado, os indicadores autorais podem apresentar valores inflacionados, refletindo mais o perfil dos membros engajados do que o panorama real e completo da produtividade acadêmica da área^(9)^.

Entende-se que a análise de medidas quantitativas de impacto científico no nível do autor, no campo da Fonoaudiologia, é justificada. A motivação pode variar conforme o contexto: seja um docente individual analisando sua própria produção ou a de outrem; um administrador tomando decisões sobre contratação, estabilidade ou promoção; ou uma agência de fomento avaliando pedidos de financiamento^(9)^. A *ResearchGate* transcende fronteiras geográficas, permitindo que pesquisadores se conectem e colaborem com colegas ao redor do mundo. Isso promove esforços de pesquisa internacionais, colaboração interdisciplinar e a troca de boas práticas e ideias inovadoras^(11)^. De qualquer forma, certamente será necessário promover ações de conscientização e capacitação para que os pesquisadores compreendam os desafios relacionados ao uso das redes sociais e os motivos pelos quais devem utilizá-las^(15)^. Nesse cenário, destaca-se o uso estratégico da *ResearchGate*, cuja interface voltada à comunidade científica permite não apenas a ampliação da visibilidade das produções em Fonoaudiologia, mas também o fortalecimento de colaborações, a disseminação do conhecimento em tempo real e a construção de indicadores alternativos de impacto. A exploração dessa rede pode contribuir para uma cultura científica mais aberta, conectada e alinhada com as demandas contemporâneas de divulgação e acessibilidade da ciência.
